# Prognostic significance of six clinicopathological features for biochemical recurrence after radical prostatectomy: a systematic review and meta-analysis

**DOI:** 10.18632/oncotarget.22459

**Published:** 2017-11-06

**Authors:** Haoran Liu, Hui Zhou, Libin Yan, Tao Ye, Hongyan Lu, Xifeng Sun, Zhangqun Ye, Hua Xu

**Affiliations:** ^1^ Department of Urology, Tongji Hospital, Tongji Medical College, Huazhong University of Science and Technology, Wuhan 430030, China; ^2^ Institute of Urology, Tongji Hospital, Tongji Medical College, Huazhong University of Science and Technology, Wuhan 430030, China

**Keywords:** clinicopathological features, biochemical recurrence, prostate cancer, radical prostatectomy, meta-analysis

## Abstract

Identifying patients with high risk of biochemical recurrence after radical prostatectomy is of immense value in clinical practice. Assessment of prognostic significance of specific clinicopathological features plays an important role in surgical management after prostatectomy. The purpose of our meta-analysis was to investigate the association between the six pathological characteristics and the prognosis of prostate cancer. We carried out a systematic document retrieval in electronic databases to sort out appropriate studies. Outcomes of interest were gathered from studies comparing biochemical recurrence-free survival (BCFS) in patients with the six pathological traits. Studies results were pooled, and hazard ratios (HRs) combined with corresponding 95% confidence intervals (CIs) for survival were used to estimate the effect size. 29 studies (21,683 patients) were enrolled in our meta-analysis. All the six predictors were statistically significant for BCFS with regard to seminal vesicle invasion (HR = 1.97, 95% CI = 1.79–2.18, *p* < 0.00001), positive surgical margin (HR = 1.79, 95% CI = 1.56–2.06, *p* < 0.00001), extracapsular extension (HR = 2.03, 95% CI = 1.65–2.50, *p* < 0.0001), lymphovascular invasion (HR = 1.85, 95% CI = 1.54–2.22, *p* < 0.00001), lymph node involvement (HR = 1.88, 95% CI = 1.37–2.60, *p* = 0.0001) and perineural invasion (HR = 1.59, 95% CI = 1.33–1.91, *p* < 0.00001). Subgroup analysis showed that all the six predictors had significantly relationship with poor BCFS. The pooled results demonstrated that the six clinical findings indicated a worse prognosis in patients with prostate cancer. In conclusion, our results show several clinicopathological characteristics can predict the risk of biochemical recurrence after radical prostatectomy. Prospective studies are needed to further confirm the predictive value of these features for the prognosis of prostate cancer patients after radical prostatectomy.

## INTRODUCTION

As one of the most common cancers for men around the world, prostate cancer causes great mortality and morbidity to patients [1]. Although a large proportion of these patients suffer low-risk, relatively indolent tumors that have little chance to progress or require surgical treatment, quite a few prostate cancers present with high-risk tumor characteristics. Clinically localized prostate cancers are best managed by radical prostatectomy (RP), while the advanced diseases always miss the opportunities and have to undergo the androgen-deprivation therapy or other adjuvant therapies [2, 3]. However, there is considerable possibility of disease recurrence after RP [4]. Thus, identifying patients with high risk of recurrence after RP is of immense value in clinical practice, so the prognosis of patients can be evaluated better and the illness can be managed timely and effectively. PSA-defined biochemical recurrence (BCR) always antedates clinical detectable distal metastasis and cancer specific mortality, providing the opportunity to access the optimal timing for salvage treatment modalities. Accurate prediction of biochemical recurrence can thus facilitate clinical decision making for prostate cancer patients [5]. Several clinicopathological factors are well established for predicting the BCR and clinical progression, including preoperative serum PSA, biopsy Gleason score, and clinical pathological stage. Some associated predictive tools which rely on certain preoperative variables are also developed to better evaluate the BCR, metastatic progression and mortality. However, these predictive variables can only provide an approximation of disease severity, there is still doubting whether additional variables can integrate with well-established ones to increase the reliability of current prognostic tools. Meticulous clinicopathological examinations will be done for resected tissue samples after RP, offering us a systematic evaluation of the characteristics of tumor. It is an intriguing problem if we can utilize certain histopathologic findings to better predict the BCR of prostate cancer patients after RP.

One of the main challenges for urologists and pathologists is the identification of potential prognostic factors in RP specimens to predict cancer progression and mortality. Apart from conventional indicators such as tumor volume, histological type, pathological Gleason score, and TNM stage, some microscopic evaluated factors are not sufficiently studied to demonstrate their prognostic value. Prostate cancer cells can invade the muscular wall of the extraprostatic part of the seminal vesicle, periprostatic fat, surrounding perineural sheath, lymph nodes, or vessel invasion (lymphatic or venous), resulting in seminal vesicle invasion (SVI), extracapsular extension (ECE), perineural involvement (PNI), lymph node involvement (LNI), or lymphovascular invasion (LVI) respectively [6, 7]. Positive surgical margin (PSM) is defined as neoplastic cells existed at the inked margin. The detection of SVI, ECE, PNI, LNI, LVI or PSM always indicates the expansion and dissemination of carcinoma, and may accompany with dismal results and high biochemical recurrence (BCR) incidence [8]. Whether the six clinicopathological features can become significant predictors of prognosis of prostate cancer after RP is a precious exploration. In order to identify more predictive factors from pathological evaluation to facilitate the clinical management, we performed this systematic review and meta-analysis of published papers to explore these specific features and their impacts on the BCR of prostate cancer after RP.

## RESULTS

### Study selection and characteristics

**Figure 1:**
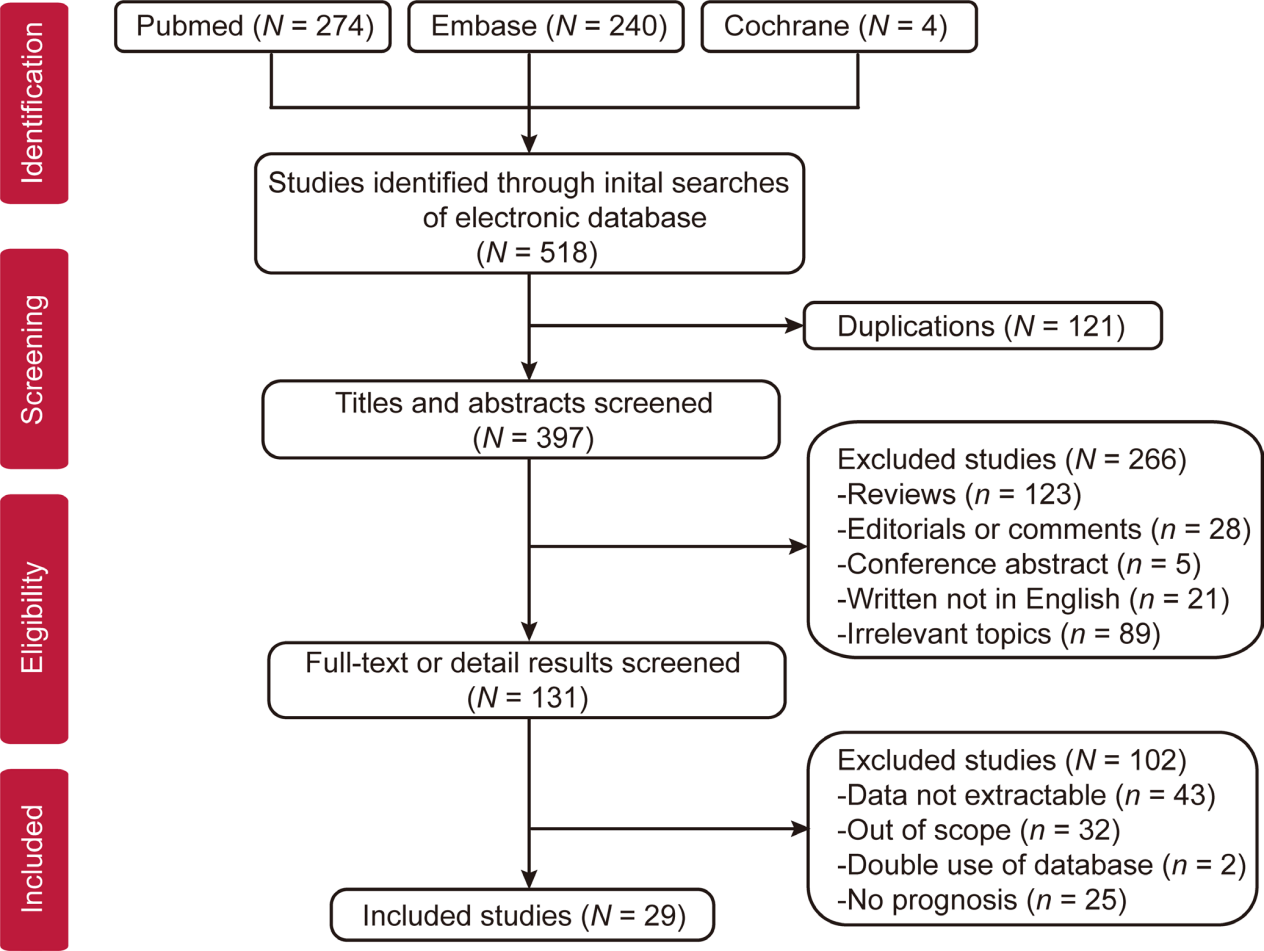
Flow diagram of studies selection

Based on the searching strategies, a total of 518 studies (274 from Pubmed, 240 from Embase, 4 from cochrane, 0 from CNKI) were screening to determine their eligibility. 121 overlapped studies were excluded and there were 397 studies left. After carefully reviewed the titles and abstracts of all identified studies, we abnegated 266 articles whose types did not meet our requirements, such as reviews, editorials, comments, non-English articles or irrelevant topics. Eventually, 29 full publications met our eligibility criteria [6, 7, 9–35]. The PRISMA study selection diagram is shown in Figure 1.

The majority of identified studies examined the prognostic value of SVI, PSM, ECE, LVI, LNI, PIN on prostate cancer prognosis. The details of each study design and clinicopathological characteristics can be seen in Table 1, and pathological findings in each studies could be seen in Supplementary Table 1. The histopathological examination was done on the specimens from resected tumors. Most of the studies used the biochemical recurrence free survival (BCFS) rates to evaluate the prognostic value of six clinicopathological indicators to survival, and then BCFS was used as a common endpoint for further evaluation.

**Table:** 

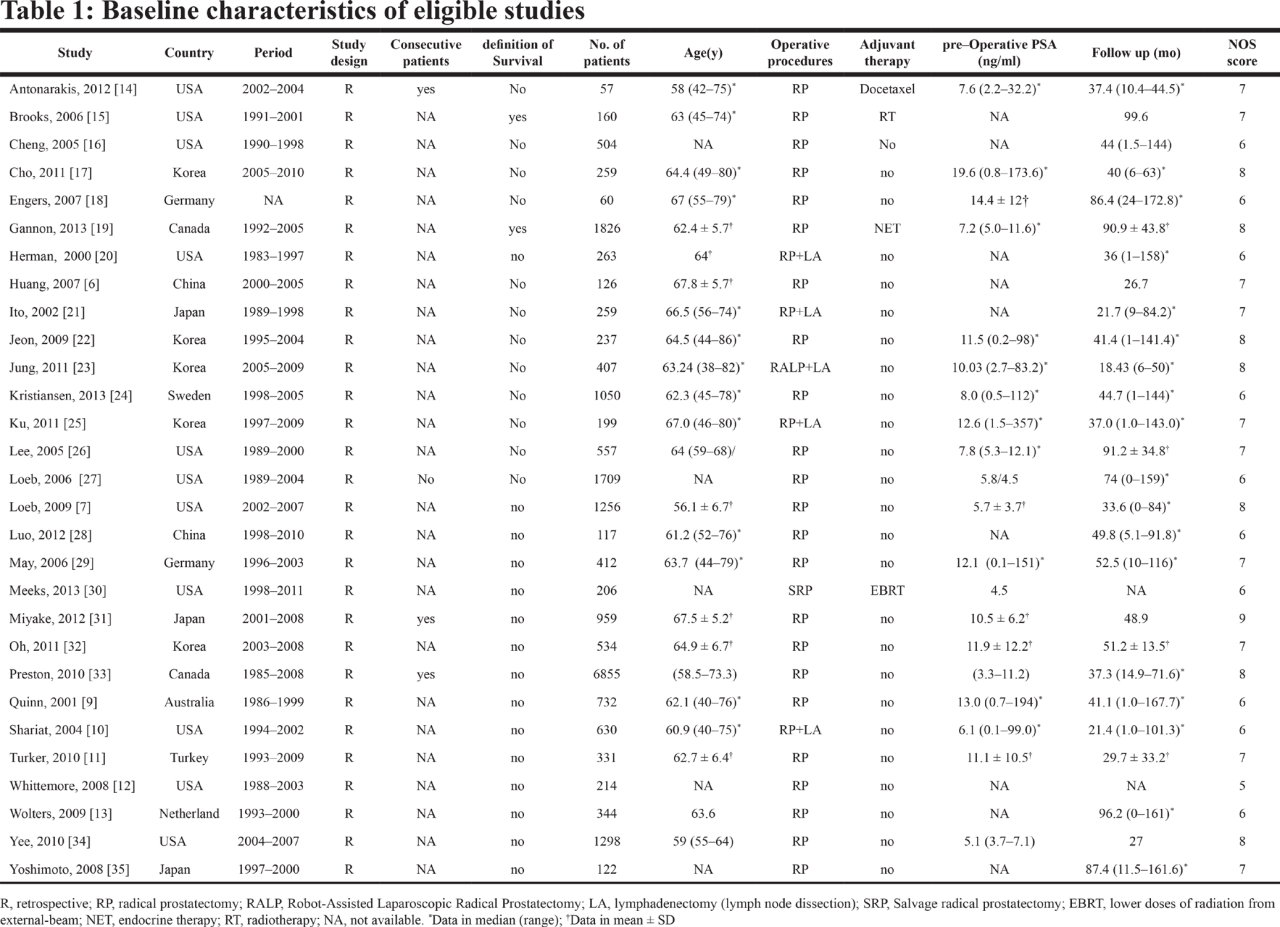

### Prognostic values of six clinicopathological features in prostate cancer after RP

**Figure 2:**
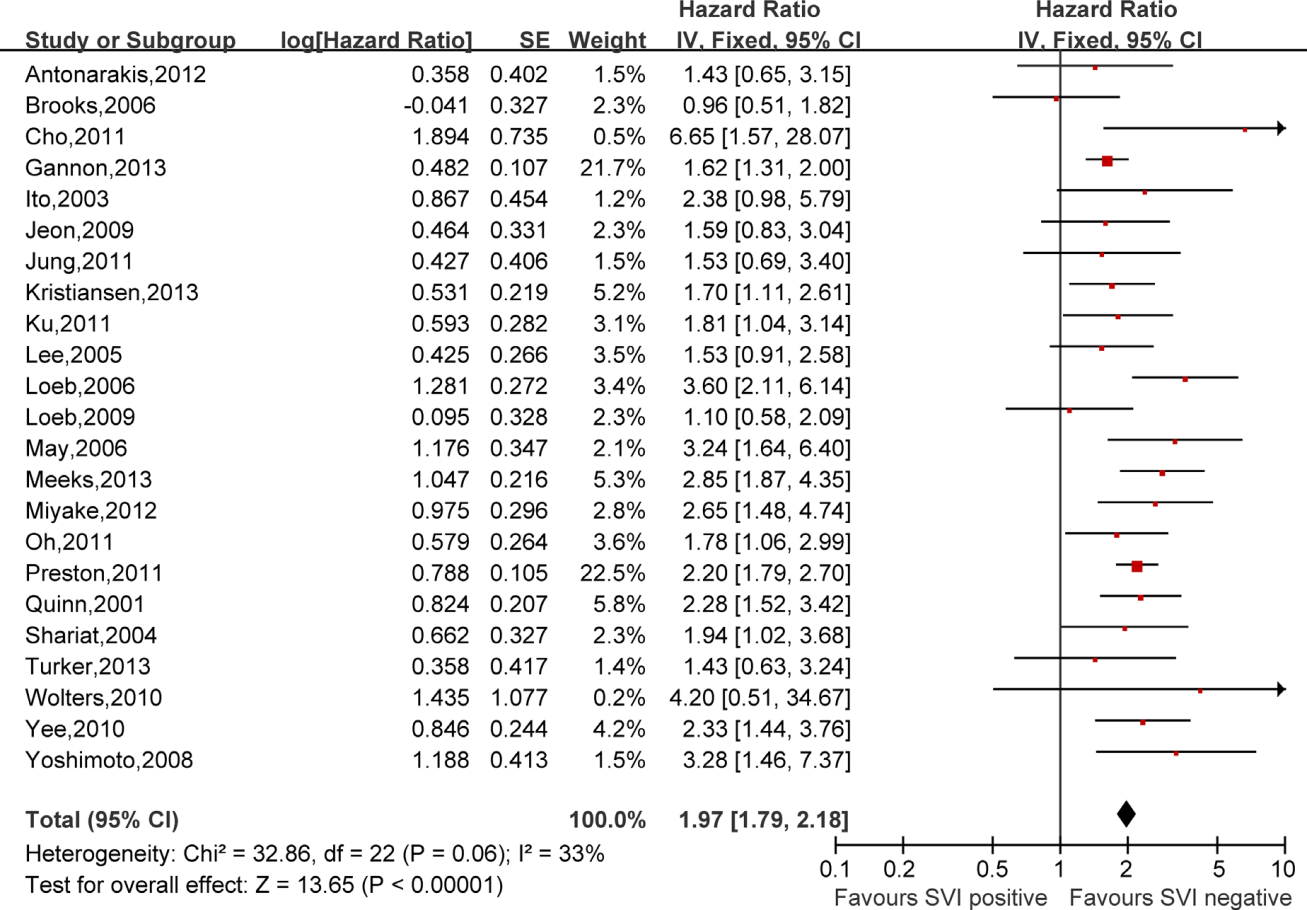
Meta-analysis of the prognostic values of SVI in prostate cancer after RP

**Figure 3:**
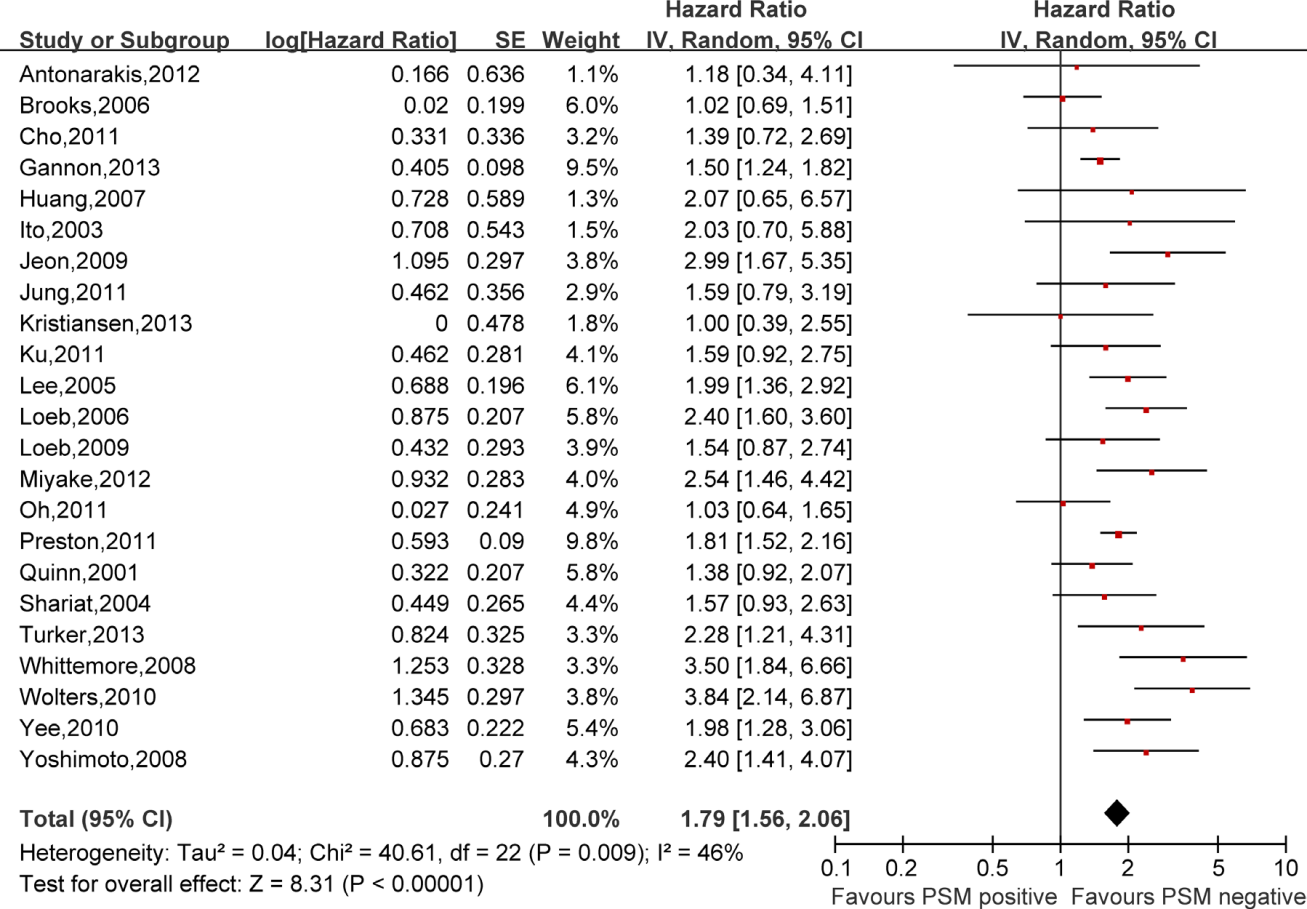
Meta-analysis of the prognostic values of PSM in prostate cancer after RP

**Figure 4:**
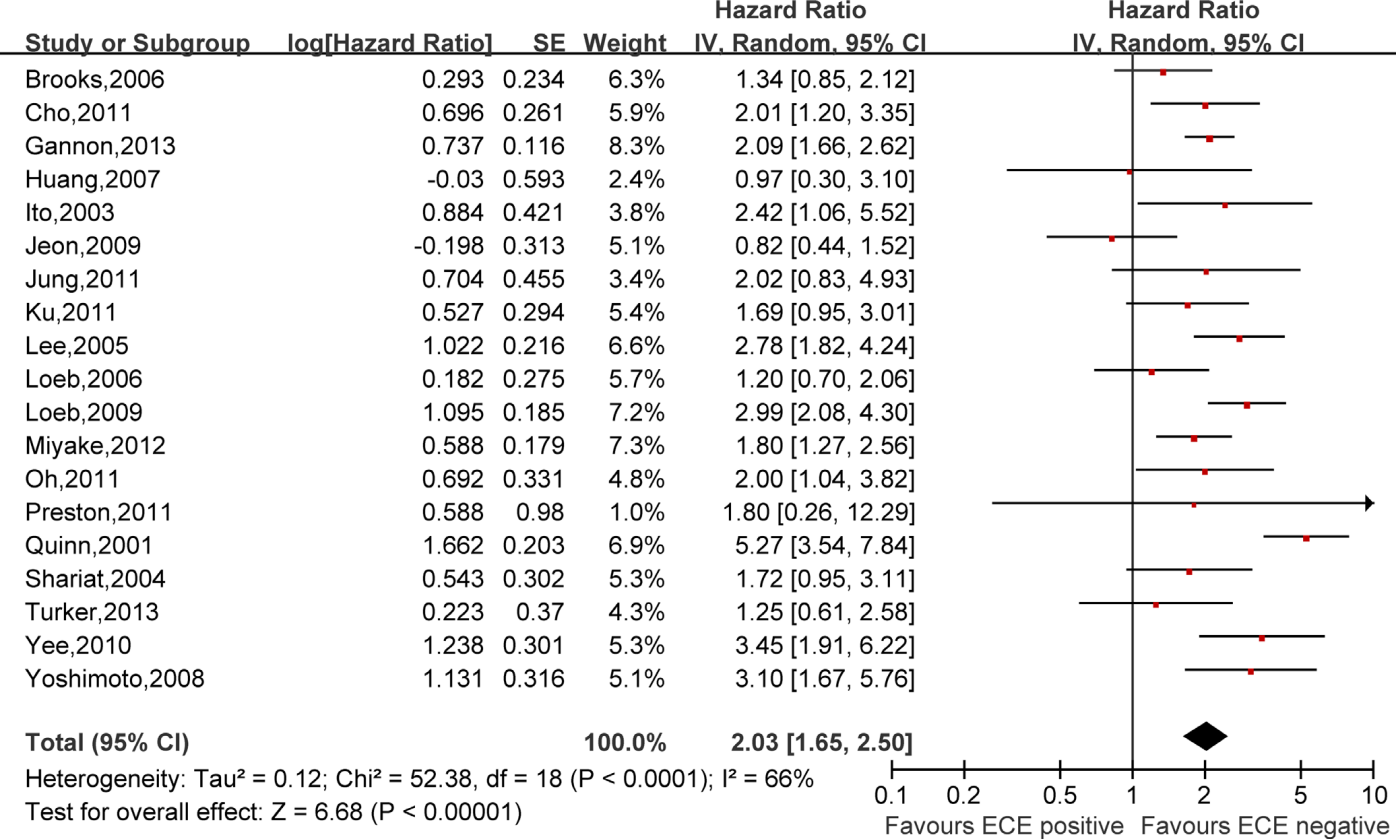
Meta-analysis of the prognostic values of ECE in prostate cancer after RP

**Figure 5:**
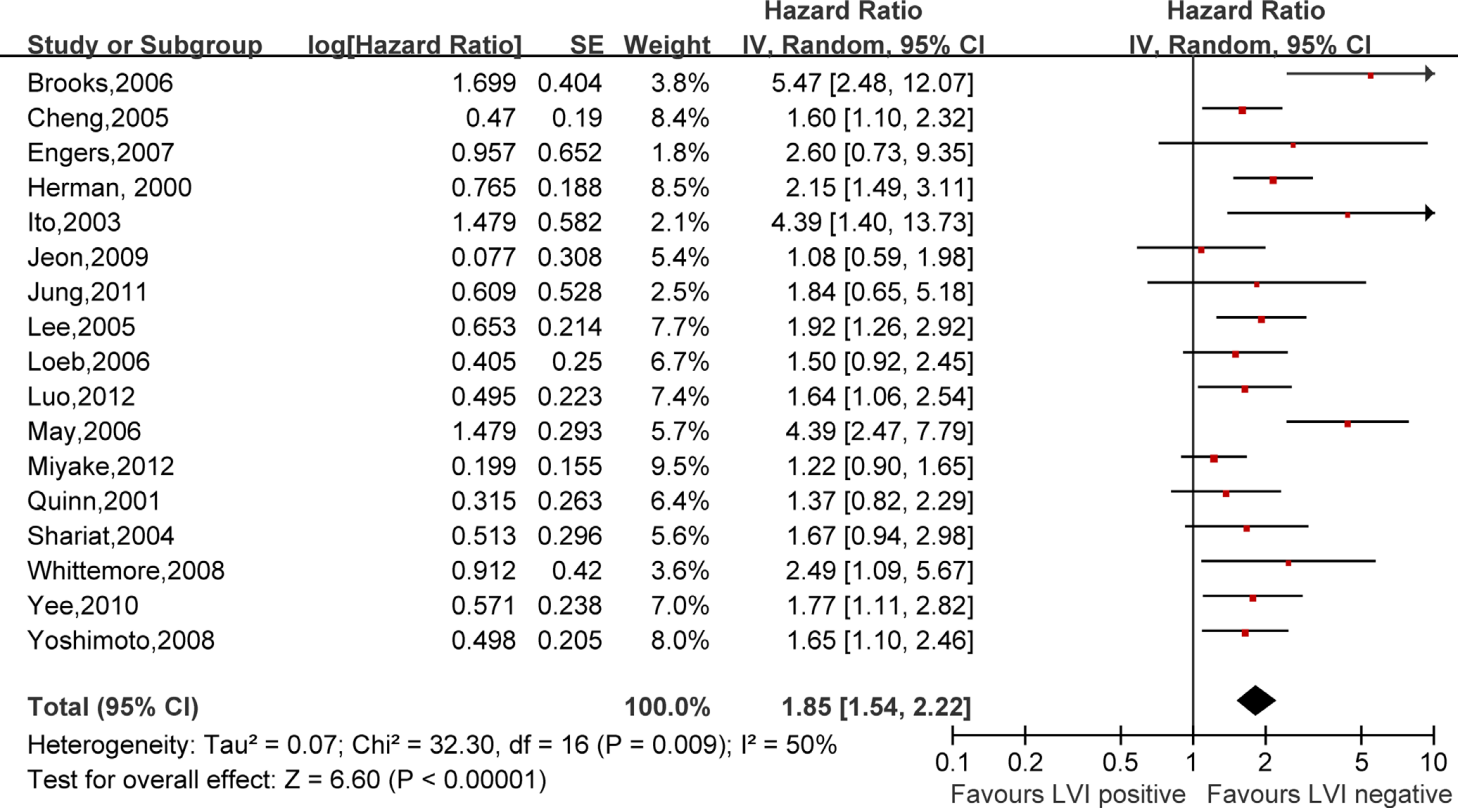
Meta-analysis of the prognostic values of LVI in prostate cancer after RP

**Figure 6:**
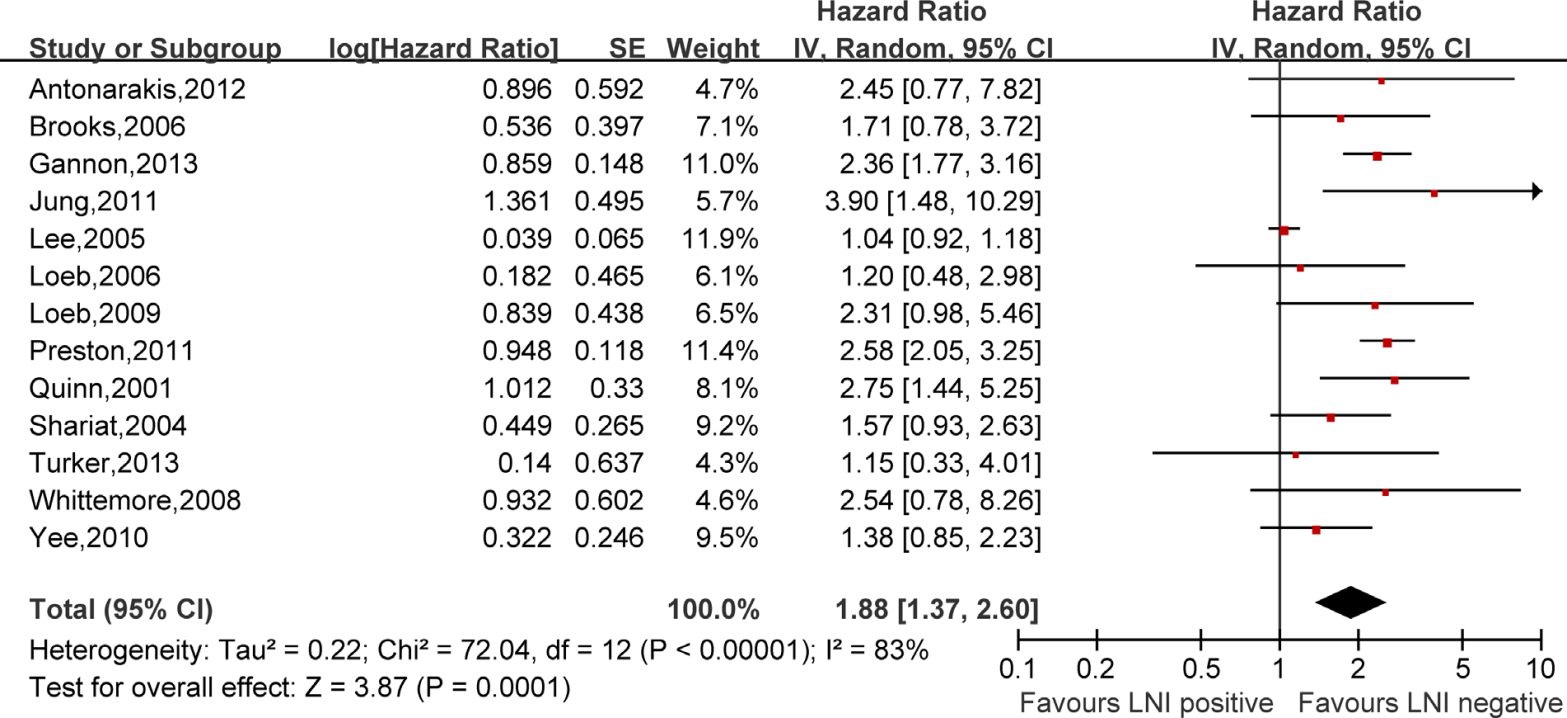
Meta-analysis of the prognostic values of LNI in prostate cancer after RP

**Figure 7:**
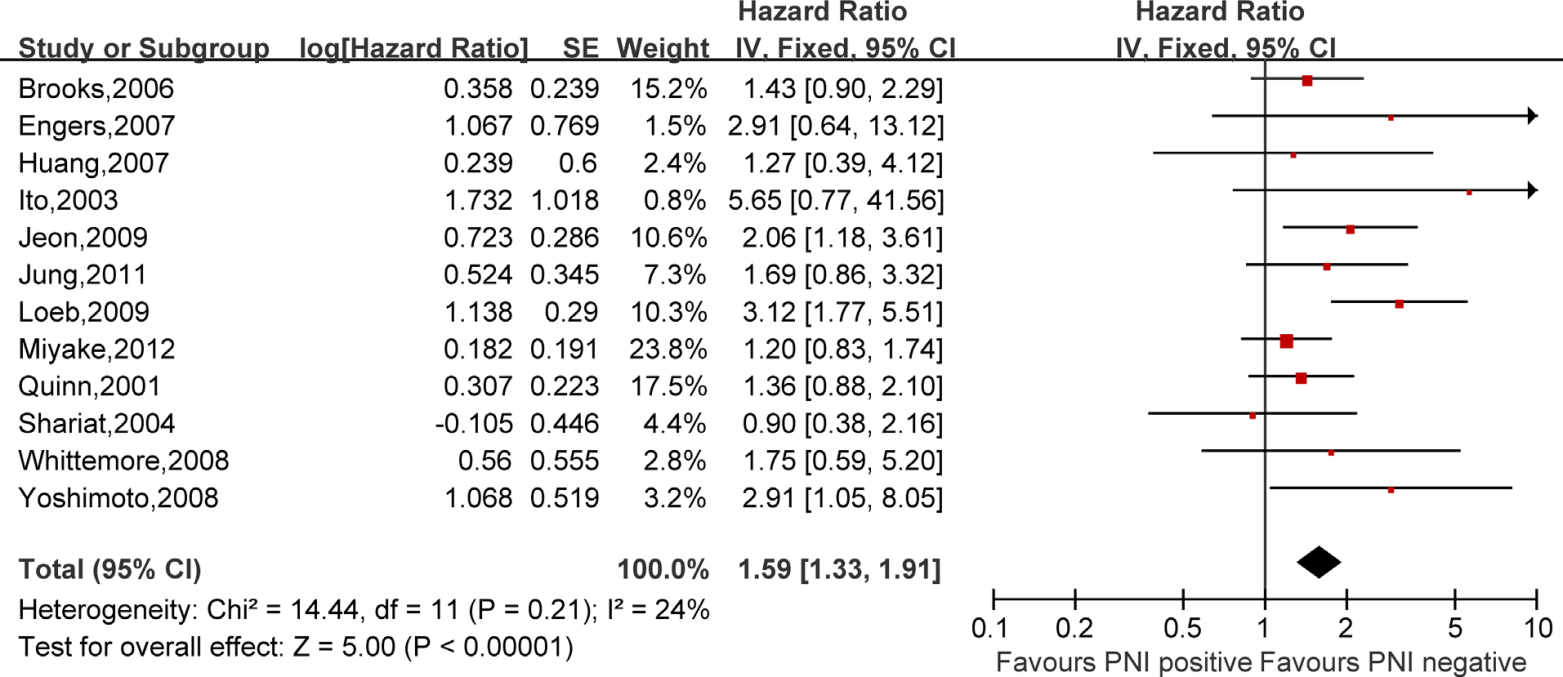
Meta-analysis of the prognostic values of PNI in prostate cancer after RP

Hazard ratios (HRs) and 95% confidence intervals (CIs) respectively of univariate and multivariate Cox proportional hazard regression models of 29 literatures were listed in Supplementary Table 1. Meanwhile, the pooled HRs and 95% CIs for each clinicopathological feature are listed in Table 2. Our pooled results showed that all the six predictors were statistically significant for BCFS, the total analyses results were as follows: SVI (pooled HR:1.97 (1.79–2.18), *p* < 0.00001, Figure 2), PSM (pooled HR:1.79 (1.56–2.06), *p* < 0.00001, Figure 3), ECE (pooled HR: 2.03 (1.65–2.50), *p* < 0.0001, Figure 4), LVI (pooled HR:1.85 (1.54–2.22), *p* < 0.00001, Figure 5), LNI (pooled HR: 1.88, (1.37–2.60), *p* = 0.0001, Figure 6), PNI (pooled HR:1.59 (1.33–1.91), *p* < 0.00001, Figure 7).

**Table 2:** Overall analyses of pathological factors in biochemical recurrence in prostate cancer after radical prostatectomy

Outcome of interest	No. of studies	HR (95% CI)	*p*-value	Study heterogeneity				Effect Model
				Chi^2^	df	*I*^2^	*p*-value	
SVI								
Total Analysis	1,2,4,6,9–16,18–25,29	1.97 [1.79, 2.18]	**< 0.00001**	32.86	22	33%	0.06	Fixed
Univariate Analysis	1,2,4,6, 9–12,16,19,20,23,24,27,29	3.61 [2.80, 4.66]	**< 0.00001**	48.04	13	73%	**< 0.00001**	Random
Multivariate Analysis	1,2,4,6,10,11,13–16,18, 20–25,27,28	1.93 [1.73, 2.14]	**< 0.00001**	27.61	18	35%	0.07	Fixed
PSM								
Total Analysis	1–3,6,8–16,20–29	1.79 [1.56, 2.06]	**< 0.00001**	40.61	22	46%	**0.009**	Random
Univariate Analysis	1,2,4,6,9–12,16,20,23,24,27,29	2.34 [2.09, 2.63]	**< 0.00001**	46.60	13	72%	**< 0.0001**	Random
Multivariate Analysis	1,3,6,8,10,11,13–16,20–28	1.91 [1.66, 2.20]	**< 0.00001**	32.71	18	45%	**0.02**	Random
ECE								
Total Analysis	2,4,6,8–11,13–16,20–25,28,29	2.03 [1.65, 2.50]	**< 0.00001**	52.38	18	66%	**< 0.0001**	Random
Univariate Analysis	2,4,6,9–11,16,20,23,24,29	3.44 [2.63, 4.52]	**< 0.00001**	39.49	10	75%	**< 0.0001**	Random
Multivariate Analysis	4,6,8–11,13–16, 20–22,24,25,28	1.93 [1.61, 2.31]	**< 0.00001**	26.24	15	43%	**0.04**	Random
LVI								
Total Analysis	2,3,5,7,9,10,11,14,15,17,18,20,23,24,26,28,29	1.85 [1.54, 2.22]	**< 0.00001**	32.30	16	50%	**0.009**	Random
Univariate Analysis	2,5,7,9,10,11,17,20,23,24	2.73 [1.90, 3.94]	**< 0.00001**	41.34	9	78%	**< 0.00001**	Random
Multivariate Analysis	2,3,9,10,14,15,18,20,23,24,26,28,29	1.85 [1.48, 2.33]	**< 0.00001**	30.48	12	61%	**0.002**	Random
LNI								
Total Analysis	1,2,6,11,14–16,22–26,28	1.88 [1.37, 2.60]	**0.0001**	72.04	12	83%	**< 0.00001**	Random
Univariate Analysis	1,2,6,11,16,23,24	6.09 [3.29, 11.27]	**< 0.00001**	47.31	6	87%	**< 0.00001**	Random
Multivariate Analysis	1,2,6,11,14–16,22–26,28	1.88 [1.37, 2.60]	**0.0001**	72.04	12	83%	**< 0.00001**	Random
PIN								
Total	2,5,8–11,16,20,23,24,26,29	1.59 [1.33, 1.91]	**< 0.00001**	14.44	11	24%	0.21	Fixed
Univariate Analysis	2,5,9–11,16,20,23,24,29	2.29 [1.92, 2.73]	**< 0.00001**	7.72	9	0%	0.56	Fixed
Multivariate Analysis	8,10,11,20,23,24,26	1.39 [1.12, 1.74]	**0.003**	3.96	6	0%	0.68	Fixed

CI = Confidence interval; HR = hazard ratio; LVI = lymphovascular invasion; PNI = perineural invasion; SVI = seminal vesicle invasion; ECE = extracapular extension; LNI = lymph node involvement; PSM = positive surgical margin.

^*^Statistically significant results are shown in bold.

### Study heterogeneity and publication bias

Heterogeneity was assessed mainly by forest plots and I^2^ statistics. Inspection of forest plots did not reveal substantial heterogeneity. In our meta-analysis, investigation of publication bias by funnel plots showed no substantial funnel plot asymmetry for prognosis of six predictors, suggesting no presence of significant publication or selection bias. The details were shown in Supplementary Figure 1A–1F.

### Subgroup analysis

We also underwent the subgroup analyses which categorized by univariate and multivariate analysis; primary and second endpoint; area; year of publication; sample size. The pooled HRs and 95% CIs were compared in subgroups (Table 3). HRs results of univariate models were much higher than multivariate ones, because the multivariate analyses could include more influencing factors. If the clinicopathologic factor was the primary endpoint of a study, its HR results were uncertain to be higher than the second endpoint counterpart. Prognostic values had no obvious difference between eastern and western, between publication year and the sample size of studies.

**Table 3:** Subgroup analysis

	LVI	PNI	SVI	ECE	LNI	PSM
Overall	1.85 [1.54, 2.22]	1.59 [1.33, 1.91]	1.97 [1.79, 2.18]	2.03 [1.65, 2.50]	1.88 [1.37, 2.60]	1.79 [1.56, 2.06]
HR analysis						
Univariate	2.73 [1.90, 3.94]	2.29 [1.92, 2.73]	3.61 [2.80, 4.66]	3.44 [2.63, 4.52]	6.09 [3.29, 11.27]	2.34 [2.09, 2.63]
Multivariate	1.85 [1.48, 2.33]	1.39 [1.12, 1.74]	1.93 [1.73, 2.14]	1.95 [1.61, 2.36]	1.88 [1.37, 2.60]	1.91 [1.66, 2.20]
Endpoint						
Primary endpoint	2.05 [1.64, 2.58]	2.28 [1.62, 3.21]	2.21 [1.63, 2.99]	1.80 [0.26, 12.29]	/	/
Second endpoint	1.44 [1.18, 1.76]	1.38 [1.12, 1.72]	1.95 [1.76, 2.16]	2.03 [1.65, 2.51]	1.88 [1.37, 2.60]	1.79 [1.56, 2.06]
Area						
Eastern	1.46 [1.24, 1.72]	1.50 [1.20, 1.88]	2.13 [1.73, 2.62]	2.03 [1.41, 2.92]	3.06 [1.79, 5.25]	1.91 [1.45, 2.52]
Western	2.26 [1.74, 2.93]	1.79 [1.31, 2.44]	1.93 [1.73, 2.16]	2.02 [1.58, 2.58]	1.73 [1.23, 2.43]	1.69 [1.53, 1.87]
Year of publication						
≥ 2010	1.44 [1.16, 1.79]	1.85 [1.19, 2.87]	1.96 [1.75, 2.20]	2.01 [1.72, 2.34]	2.33 [1.98, 2.74]	1.70 [1.53, 1.89]
< 2010	2.00 [1.59, 2.50]	1.49 [1.14, 1.94]	1.99 [1.50, 2.63]	2.02 [1.38, 2.96]	1.62 [1.12, 2.35]	1.82 [1.56, 2.12]
Sample						
> 300	1.72 [1.37, 2.16]	1.48 [1.18, 1.86]	1.96 [1.76, 2.18]	2.29 [1.75, 2.99]	1.44 [1.30, 1.58]	1.71 [1.55, 1.89]
≤ 300	1.95 [1.60, 2.37]	1.81 [1.34, 2.45]	2.04 [1.61, 2.57]	1.75 [1.27, 2.40]	2.04 [1.16, 3.60]	1.94 [1.43, 2.62]

LVI = lymphovascular invasion; PNI = perineural invasion; SVI = seminal vesicle invasion; ECE = extracapular extension; LNI = lymph node involvement; PSM = positive surgical margin.

## DISCUSSION

Although clinically localized disease can be managed by RP, about 20–30% of prostate cancer patients may suffer from recurrence which can be detected by rising serum PSA initially. Detectable serum PSA after RP or rising PSA level after PSA detection absence is defined as BCR, which often precedes clinical progression and prostate cancer specific mortality [5, 8]. High risks of BCR rationalizes the administration of early salvage therapies and more frequent follow-up for patients at lower PSA levels [36]. However, the probability of BCR after RP is difficult to determine because it may vary according to several baseline risk characteristics and various tumor characteristics. Some traditional clinicopathologic features such as preoperative PSA level, biopsy Gleason score (GS) and tumor staging also show considerable prognostic predictive value and facilitate clinical decision-making by taking part in certain nomograms. One of the commonly used preoperative models for prediction of BCR is D’Amico risk stratification scheme which utilizes PSA level, biopsy GS and AJCC T stage [37]. Another predictive model designed by Stephenson et al. evaluates preoperative PSA, clinical stage, biopsy GS, year of surgery, and biopsy cores to determine the risk of BCR after RP [38]. Although these predictive tools have been externally validated, their ability for prediction of BCR needs improvement further. Routine histopathological examination of resected tumor specimens can provide us extensive details which prostate biopsy or biochemical tests can’t. Their predictive potential is also underestimated and neglected in significant measure [39]. It is still unresolved whether certain tumor characteristics can act as accurate, flexible, and easily accessible factors to assist BCR prediction.

The objective of our meta-analysis was to elucidate the association between dismal clinical detections and biochemical recurrence of prostate cancer after RP. Currently, certain predictive tools are available to predict several survival endpoints in prostate cancer including BCR and some of them have already shown considerable accuracy for predicting risks of progression, recurrence and mortality [40, 41]. However, there exist some limitations which compromise the predictive value. First, some commonly used predictive models like D’Amico risk stratification scheme, the Cancer of the Prostate Risk Assessment (CAPRA) score, and the Stephenson nomogram utilized preoperative variables to obtain the possibility of BCR after RP [40, 42]. Although some clinicopathological features from preoperative biopsy are included in these nomograms, systemic postoperative pathologic examination is capable of providing us more information with more details. Second, some prognostic variables used in these nomograms were simply proved to be significant prognostic factors with Cox proportional hazards models. Further validation of a larger population and pooled predictive value of different cohorts are needed to determine their roles in quantitative manners. In an attempt to settle these limitations, we conducted this meta-analysis to pool relevant studies and figure out the predictive values of several clinicopathological characteristics of postoperative resected specimens for prediction of BCR.

This meta-analysis combined the results from 29 studies of 21,683 patients and revealed that identification of SVI, ECE, PSM, PNI, LNI or LVI could all significantly predict high risks of prostate cancer patients, with the pooled HRs ranging from 1.59 to 2.03 (all P-value ≤ 0.0001). Hence, we observed that these pathologic findings were independent risk factors of biochemical recurrence in prostate cancer in this comprehensive meta-analysis. Our results were relatively reliable due to certain advantages. First, number of studies were sufficient and sample sizes were relatively large even after rigorous evaluation and selection of eligible studies. Second, no obvious heterogeneity and publication bias between the studies were found, subgroup analyses were also included to obviate potential confounding factor. Third, we chose a panel of clinicopathological variables which were recommended to report regularly in postoperative setting. These variables jointly were a reflection of tumor expansion and invasiveness. However, we must acknowledge that there existed potential intrinsic weaknesses in these trials contained in our meta-analysis. Most studies included in our meta-analysis were retrospective observations, without any prospective random controlled trials. Besides, due to limited data on effect of six predictors on tumor metastasis and mortality, a single survival endpoint BCR was adopted. This might limit the overall significance of our pooled results. Last, in the overall analysis of pooled HRs and 95% CIs, we accepted the outcomes of univariate Cox models when the multivariate ones were absent, so the final predictive effects could be affected by the less reliable results of univariate analyses.

In conclusion, our pooled results prove that all the six clinicopathological features we discussed can predict early BCR in patients with prostate cancer after RP. Tumor characteristics are promising prognostic factors for BCR prediction. The clinicopathological findings may be integrated into a comprehensive, reliable, and handy predictive tool with strong ability to predict patient outcome after surgical resection. Further well-designed prospective studies will offer more reliable conclusions on the predictive value of certain postoperative pathological characteristics for BCR in prostate cancer patients.

## MATERIALS AND METHODS

All methods for this systematic review and meta-analysis are outlined in a prospectively registered protocol available online (PROSPERO identifier CRD42017057810), and reporting follows PRISMA (Preferred Reporting Items for Systematic Reviews and Meta-Analyses) guidelines.

### Eligibility criteria

Eligible studies include randomised, controlled trials and nonrandomised studies (observational, cohort) that have investigated the treatment with radical prostatectomy, in participants over 36 years old with prostate cancer. Studies must have reported data on at least one of SVI, PSM, ECE, LVI, LNI and PNI.

### Search strategy

We searched published studies indexed in PubMed, Embase, Cochrane Library and the China National Knowledge Infrastructure databases until Oct 31, 2016. The search query are as follow: ((‘SVI’ or ‘ECE’ or ‘PSM’ or ‘PNI’ or ‘LNI’ or ‘LVI’) and (‘biochemical recurrence’) and (‘prostate cancer’ or ‘prostate carcinoma’ or ‘prostatic carcinoma’) and (‘radical prostatectomy’)). All the eligible papers were published in English. Following the literature search, all duplicates were excluded. References from review articles, commentaries, editorials, included studies, and conference publications of relevant medical societies were reviewed and cross-referenced to ensure completeness. Conference abstracts were excluded.

### Study selection

Studies were included if they satisfied all the following requirements according to the PICOS criteria: (1) included paitents who were pathologically confirmed with prostate cancer patients and received radical prostatectomy; (2) for intervention, radical prostatectomy, either laparoscopic radical prostatectomies or robotic assisted radical prostatectomies was performed; (3) outcomes at least reported BCFS in prostate cancer patients after RP. (4) clinicopathological features assessing prognosis at least included one of SVI, PSM, ECE, LVI, LNI and PNI. (5) Results must report the sample size, hazard ratios (HR) combined with 95% confidence intervals; (6) studies had to be original articles.

Studies were excluded if any of the following criteria were met: (1) review articles, guidelines, consensus statements, letters, editorials, and conference abstracts; (2) studies with overlapping patient population; (3) studies which didn’t provide enough data for HR and standard error (SE) estimation; (4) non-English paper.

Two reviewers (L.H.R. and Z.H.) independently evaluated the eligibility of the selected studies from the literature. Disagreements were resolved by consensus via discussion with a third reviewer (Y.Z.Q.).

### Data extraction and quality assessment

For each of eligible study, information was extracted and cross-checked by two independent investigators: (1) baseline characteristics: information of first author, year of publication, study design type, study location, period of recruitment, survival definition, sample size, age, operative procedures, adjuvant therapies, pre-operative PSA level, duration of follow-up; (2) survival analysis: end-point, patient numbers and percentage of specific pathological traits, hazard ratios (HR) combined with 95% confidence intervals (CI) and *P* value of univariate and multivariate analysis, primary endpoint and co-factors of Cox’s proportional hazards regression models.

Newcastle–Ottawa quality assessment scale for all eligible studies was used to evaluate methodological quality. Data extraction and quality assessment were performed independently by two reviewers (Y.T. and L.H.Y.). Discrepancies between the two inquirers in data extraction were resolved by discussion and consultation with a consensus (Y.Z.Q.) to reach a consensus.

### Outcomes of interest and survival end-points

The primary outcome measures used in articles were the biochemical recurrence-free survival rate (BCFS). Actually, the progression-free survival (PFS) and disease-free survival (DFS) used in specific studies were always equivalent to BCFS with the same definition of survival. For the convenience of studying, we chose BCFS as the single survival end-points. For assessing BCFS, recurrence of the disease was referred to BCR, most often regarded as postoperative serum PSA at least 0.2 ng/mL.

### HR pooled and statistical analysis

HRs and 95% CIs were employed to assess the predictive significance of six clinicopathological events on BCFS of the sick people. A pooled HR > 1 indicated a worse survival of positive group compared to the negative group. For studies which did not report HRs directly, figures and data in primary papers were employed to work out the HRs based on the methods proposed by Tiernry [43].

We employed Review Manager Software (RevMan 5.3, Cochrane Collaboration, Oxford, UK) to make our meta-analysis. Total analyses of specific pathological factors were conducted using results of multivariate models and univariate results when the multivariate ones were not available.

### Assessment of heterogeneity

We employed a chi^2^-based test for assessment of homogeneity and inconsistency index (I^2^) statistic. Furthermore, we employed random effects models for each of our analyses given the identified clinical heterogeneity [44]. The heterogeneity of combined HRs were respectively evaluated with the help of graphic examination of forest plots, and funnel plots were utilized to explore any potential publication bias.

### Subgroup analysis

To wipe off the effect of other confounding factors, we performed the subgroup analysis categorized by univariate or multivariate analysis, primary or second endpoint, area location (Eastern/Western), publication year (≥ 2010/< 2010) and sample Size (> 300/≤ 300). The pooled HRs with their 95% CIs were elevated separately, and compared in subgroups. Then the heterogeneity between studies might be understood and managed better.

## SUPPLEMENTARY MATERIALS FIGURE AND TABLE





## Abbreviations

RP: radical prostatectomy
RALP: Robot-Assisted Laparoscopic Radical Prostatectomy
LA: lymphadenectomy (lymph node dissection)
LVI: lymphovascular invasion
PNI: perineural invasion
SVI: seminal vesicle invasion
ECE: extracapular extension
LNI: lymph node involvement
PSM: positive surgical margin
pre-op PSA: preoperative PSA level
GS: Gleason score
HGPIN: High-grade prostatic intraepithelial neoplasia
pT: pathologic stage
pTI: percentage of tumor involvement
EBRT: lower doses of radiation from external-beam RT
RT: radiotherapy
P: prospective
R: retrospective
CI: Confidence interval
HR: hazard ratio
NA: not available
