# Supplementary material for: Prognostic significance of six clinicopathological features for biochemical recurrence after radical prostatectomy: a systematic review and meta-analysis
Source: Oncotarget. 2017 Nov 6;9(63):32238–49. doi: 10.18632/oncotarget.22459 (PMC6114957; doi:10.18632/oncotarget.22459)
Supplement: Supplementary file 1 [file oncotarget-09-32238-s001.pdf]

Prognostic significance of six clinicopathological features for biochemical recurrence after radical prostatectomy: a systematic review and meta-analysis

SUPPLEMENTARY MATERIALS

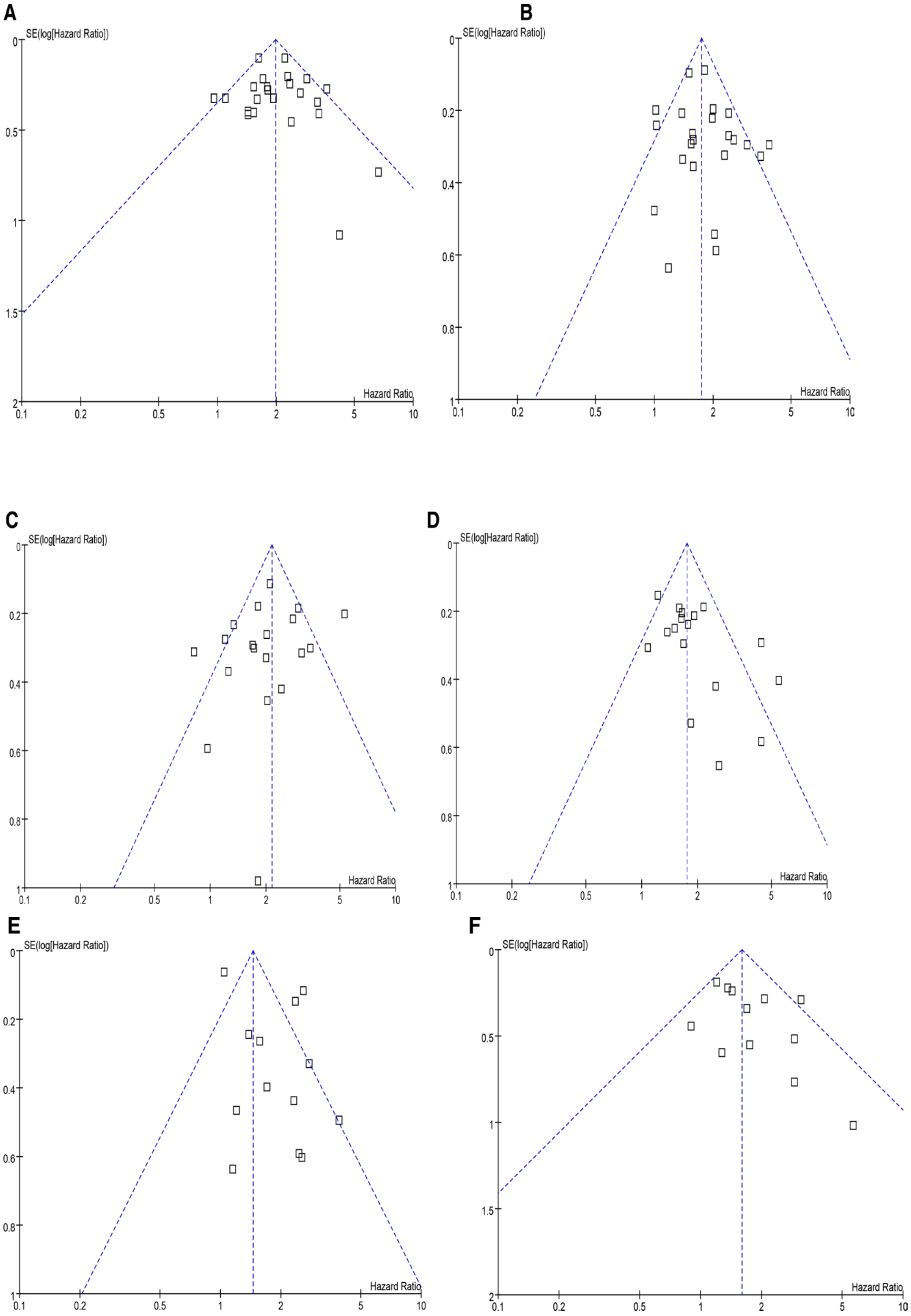

Supplementary Figure 1: (**A**) Funnel plot and meta-analysis of SVI. (**B**) Funnel plot and meta-analysis of PSM. (**C**) Funnel plot and meta-analysis of ECE. (**D**) Funnel plot and meta-analysis of LVI. (**E**) Funnel plot and meta-analysis of LNI. (**F**) Funnel plot and meta-analysis of PNI.

Supplementary Table 1: Estimation of the hazard ratio. See Supplementary_Table_1
