# Supplementary material for: Prognostic significance of six clinicopathological features for biochemical recurrence after radical prostatectomy: a systematic review and meta-analysis
Source: Oncotarget. 2017 Nov 6;9(63):32238–49. doi: 10.18632/oncotarget.22459 (PMC6114957; doi:10.18632/oncotarget.22459)
Supplement: Supplementary file 2 [file oncotarget-09-32238-s002.doc]

**Supplementary Table 1:** Estimation of the hazard ratio

| Study | Endpoint | No. of patients | No. of pathological features | Pts(%) | univariate analysis  HR (95% CI) | p-value | Multivariate analysis  HR (95% CI) | p-value | Primary  endpoint | Co-factors |
| --- | --- | --- | --- | --- | --- | --- | --- | --- | --- | --- |
| Antonarakis,2012 | SVI | 57 | 35 | 61.4 | 2.18(1.11-4.29) | 0.024 | 1.43(0.65-3.14) | 0.378 | PTEN,  MYC,  Ki67 | GS, SVI, LNI, PSM, pre-op PSA, IHC signature |
|  | LNI | 57 | 22 | 38.6 | 1.19(0.64-2.22) | 0.585 | 2.45(0.77-7.83) | 0.13 |
|  | PSM | 57 | 37 | 64.9 | 1.03(0.55-1.94) | 0.931 | 1.18(0.34-4.12) | 0.799 |
| Brooks,2006 | LVI | 160 | 18 | 11.3 | 3.54(2.0-6.1) | <0.001 | 5.47(2.5-12.2) | <0.001 | LVI | GS, LNI, PSM, pre-op PSA, SVI, ECE, PNI, Hormones during treatment, radiotherapy dose |
|  | PNI | 160 | 34 | 21.3 | 1.43(0.9-2.3) | 0.14 | NA | NA |
|  | SVI | 160 | 90 | 56 | 2.20（1.4-3.4） | <0.001 | 0.96(0.5-1.8) | 0.89 |
|  | ECE | 158 | 93 | 58.9 | 1.27(0.8-2.0) | 0.27 | NA | NA |
|  | LNI | 160 | 11 | 6.9 | 3.29(1.7-6.3) | 0.002 | 1.71(0.8-3.8) | 0.19 |
|  | PSM | 160 | 114 | 71.3 | 1.02(0.7-1.6) | 0.93 | NA | NA |
| Cheng,2005 | LVI | 504 | 106 | 21 | NA | NA | 1.6(1.12-2.36) | 0.01 | LVI | GS, vascular invasion, pT |
| Cho,2011 | SVI | 259 | 25 | 9.65 | 7.89(4.00-15.59) | <0.001 | 6.644(1.573-28.061) | 0.012 | tumor  volume | GS, SVI, PSM, pre-op PSA, ECE, prostate volume, tumor percentage |
|  | ECE | 259 | 77 | 29.7 | 4.44(2.33-8.48) | <0.001 | 2.005(1.202-2.835) | 0.048 |
|  | PSM | 259 | 79 | 30.5 | 1.393(0.721-2.694) | 0.324 | NA | NA |
| Engers,2007 | LVI | 60 | 6 | 10 | 2.604(0.725-9.346) | 0.142 | NA | NA | Rac  GTPase | GS, pre-op PSA, pT, blood vessel invasion, age, pN stage, LVI, PNI |
|  | PNI | 60 | 6 | 10 | 2.91(0.65-13.16) | 0.164 | NA | NA |
| Gannon,2013 | SVI | 1826 | 256 | 14 | 4.73(3.94-5.67) | <0.001 | 1.62(1.31-1.99) | <0.001 | NF-kB,  P65 | GS, LNI, PSM, pre-op PSA, ECE, SVI, NF-κB dichotomised variable |
|  | ECE | 1826 | 668 | 36.6 | 5.07(4.20-6.18) | <0.001 | 2.09(1.66-2.62) | <0.001 |
|  | LNI | 1826 | 68 | 6 | 7.96(6.10-10.4) | <0.001 | 2.36(1.76-3.15) | <0.001 |
|  | PSM | 1826 | 397 | 22 | 2.58(2.15-3.10) | <0.001 | 1.50(1.24-1.82) | <0.001 |
| Herman, 2000 | LVI | 263 | 91 | 35 | 2.15(1.49-3.11) | <0.001 | NA | NA | LVI | NA |
| Huang,2007 | PNI | 117 | 44 | 37.6 | NA | NA | 1.27(0.39-4.10) | 0.695 | P53,  XRCC1 | age, pre-op PSA, ECE, PSM, vascular invasion, PNI, tumor multifocality, HGPIN,GS |
|  | ECE | 117 | 44 | 37.6 | NA | NA | 0.97(0.30-3.07) | 0.951 |
|  | PSM | 117 | 45 | 35.7 | NA | NA | 2.07(0.65-6.54) | 0.217 |
| Ito,2003 | LVI | 82 | 38 | 46.3 | 6.54(2.27-18.87) | 0.0005 | 4.39(1.40-13.70) | 0.0191 | LVI | GS, ECE, PSM, SVI, PNI |
|  | PNI | 82 | 64 | 78 | 5.65(0.77-41.67) | 0.0895 | NA | NA |
|  | SVI | 82 | 7 | 8.5 | 2.38（0.96-5.68） | 0.06 | NA | NA |
|  | ECE | 82 | 32 | 39 | 2.69(1.29-5.59) | 0.0082 | 2.42(1.06-5.52) | 0.036 |
|  | PSM | 82 | 5 | 6.1 | 2.03(0.70-5.88) | 0.1925 | NA | NA |
| Jeon,2009 | LVI | 237 | 41 | 17.3 | 2.50(1.46-4.26) | 0.001 | 1.08(0.59-1.97) | 0.797 | PNI | GS, LVI, PSM, pre-op PSA, ECE, SVI |
|  | PNI | 237 | 100 | 42.2 | 2.68(1.64-4.38) | <0.001 | 2.06(1.17-3.59) | 0.011 |
|  | SVI | 237 | 32 | 13.5 | 3.55(2.08-6.07) | <0.001 | 1.59(0.83-3.04) | 0.16 |
|  | ECE | 237 | 84 | 35.4 | 2.24(1.38-3.65) | 0.001 | 0.82(0.44-1.50) | 0.52 |
|  | PSM | 237 | 86 | 36.3 | 4.14(2.51-6.84) | <0.001 | 2.99(1.67-5.36) | <0.001 |
| Jung,2011 | LVI | 407 | 27 | 6.6 | 1.839(0.654-5.172) | 0.248 | NA | NA | PNI,  LVI,  HGPIN | GS, LNI, PSM, pre-op PSA, ECE, HGPIN |
|  | PNI | 407 | 162 | 39.8 | 2.949(1.597-5.445) | 0.001 | 1.688(0.859-3.317) | 0.129 |
|  | SVI | 407 | 69 | 17 | 2.481(1.312-4.694) | 0.005 | 1.532(0.691-3.393) | 0.294 |
|  | ECE | 407 | 57 | 14 | 3.000(1.498-6.006) | 0.002 | 2.021(0.829-4.928) | 0.122 |
|  | LNI | 407 | 12 | 2.9 | 13.741(5.890-32.055) | <0.001 | 3.901(1.478-10.297) | 0.006 |
|  | PSM | 407 | 129 | 31.7 | 3.013(1.670-5.435) | <0.001 | 1.588(0.777-3.247) | 0.205 |
| Kristiansen,2013 | SVI | 1050 | 60 | 5.7 | 1.7(1.1-2.6) | 0.015 | NA | NA | SVI | PSM, Invasion of vas deferens, GS |
|  | PSM | 1050 | 17 | 1.6 | 1.0(0.4-2.6) | 0.97 | NA | NA |
| Ku,2011 | SVI | 199 | 42 | 21.1 | NA | NA | 1.809(1.041-3.145) | 0.035 | pelvic  LA | GS, PSM, ECE, SVI |
|  | ECE | 199 | 107 | 53.8 | NA | NA | 1.693(0.951-3.013) | 0.074 |
|  | PSM | 199 | 91 | 45.7 | NA | NA | 1.587(0.914-2.754) | 0.101 |
| Lee,2005 | LVI | 557 | NA | NA | NA | NA | 1.92(1.26-2.92) | <0.01 | FH | age, pre-op PSA, clinical T stage, GS, LNI, LVI, ECE, PSM, SVI,tumor volume |
|  | SVI | 557 | 46 | 8.3 | NA | NA | 1.53(0.90-2.55) | 0.11 |
|  | ECE | 557 | 184 | 33 | NA | NA | 2.78(1.83-4.27) | <0.01 |
|  | LNI | 557 | NA | NA | NA | NA | 1.04(0.91-1.17) | 0.51 |
|  | PSM | 557 | NA | NA | NA | NA | 1.99(1.35-2.91) | <0.01 |
| Loeb,2006 | LVI | 1709 | 118 | 6.9 | NA | NA | 1.5(0.9-2.4) | 0.1 | LVI | GS, PSM, ECE, LNI, SVI, LVI |
|  | SVI | 1709 | 63 | 3.7 | NA | NA | 3.6(2.1-6.1) | <0.0001 |
|  | ECE | 1709 | 251 | 14.7 | NA | NA | 1.2(0.7-1.9) | 0.6 |
|  | LNI | 1709 | 11 | 0.64 | NA | NA | 1.2(0.5-3.2) | 0.6 |
|  | PSM | 1709 | 341 | 20 | NA | NA | 2.4(1.6-3.6) | <0.0001 |
| Loeb,2009 | PNI | 1256 | 188 | 15 | 3.12(1.77-5.52) | <0.001 | NA | NA | PNI | ECE, PSM, LNI, PSA level, clinical stage, biopsy GS, nerve sparing |
|  | ECE | 1256 | 274 | 21.8 | 4.66(3.36-6.47) | <0.001 | 2.99(2.08-4.30) | <0.001 |
|  | LNI | 1256 | 29 | 2.3 | 5.65(2.68-11.91) | <0.001 | 2.29(0.97-5.40) | 0.059 |
|  | PSM | 1256 | 84 | 6.7 | 1.87(1.12-3.15) | 0.017 | 1.54(0.88-2.77) | 0.128 |
| Luo,2012 | LVI | 87 | 18 | 20.69 | 1.64(1.06-2.54) | 0.012 | NA | NA | LVI | NA |
| May,2006 | LVI | 412 | 86 | 20.9 | NA | NA | 4.39(2.47-7.80) | <0.001 | LVI | PSA density, pre-op PSA, GS, positive biopsy cores, SVI |
|  | SVI | 412 | 53 | 12.9 | NA | NA | 1.10 (0.58–2.10) | 0.776 |
| Meeks,2013 | SVI | 206 | 66 | 32 | 2.85(1.87-4.36） | <0.001 | NA | NA | SVI | NA |
| Miyake,2012 | LVI | 959 | 271 | 28.8 | 1.34(1.012-1.774) | 0.041 | 1.22(0.900-1.654) | 0.2 | Gleason  pattern | PSA, biopsy GS, ECE, PSM, lymphatic invasion, microvenous invasion, PNI |
|  | PNI | 959 | 661 | 68.9 | 1.97(1.32-2.95) | <0.001 | 1.20(0.83-1.74) | 0.34 |
|  | SVI | 959 | 72 | 7.5 | 5.10(1.93-13.45) | <0.001 | 2.65(1.48-4.74) | <0.001 |
|  | ECE | 959 | NA | NA | 3.08(1.58-6.02) | <0.001 | 1.80(1.27-2.56) | <0.001 |
|  | PSM | 959 | 355 | 37 | 3.00(1.559-5.772) | <0.001 | 2.54(1.458-4.426) | <0.001 |
| Oh,2011 | SVI | 534 | 46 | 8.6 | NA | <0.001 | 1.785(1.065-2.994) | 0.028 | pTI | age, pre-op PSA, prostate volume, pathological GS, ECE, PSM, SVI |
|  | ECE | 534 | 168 | 31.5 | NA | NA | 1.998(1.045-3.820) | 0.036 |
|  | PSM | 534 | 200 | 37.5 | NA | NA | 1.027(0.640-1.649) | 0.912 |
| Preston,2010 | SVI | 6855 | 452 | 6.6 | NA | NA | 2.20(1.79-2.70) | <0.0001 | ECE | PSM, pre-op PSA, RP date, GS, LNI, SVI |
|  | ECE | 6855 | 4122 | 60.1 | NA | NA | 1.80(1.48-2.17) | <0.0001 |
|  | LNI | 6855 | 278 | 4.1 | NA | NA | 2.58(2.05-3.25) | <0.0001 |
|  | PSM | 6855 | 1261 | 18 | NA | NA | 1.81(1.51-2.15) | <0.0001 |
| Quinn,2001 | LVI | 731 | 38 | 5.2 | 4.15(2.55-6.75) | <0.001 | 1.37(0.82-2.30) | 0.23 | PSA,  GS,  pT | pre-op PSA, RP GS, pathological stage, SVI, LNI, year of RP, PNI, vascular invasion, PSM, ECE, adjuvant therapy |
|  | PNI | 732 | 375 | 51.3 | 2.37(1.56-3.60) | <0.0001 | 1.36(0.88-2.11) | 0.17 |
|  | SVI | 732 | 96 | 13.1 | 5.30(3.78-7.45) | <0.0001 | 2.28(1.52-3.42) | <0.0001 |
|  | ECE | 732 | 155 | 21.2 | 5.27(3.54-7.84) | <0.0001 | NA | NA |
|  | LNI | 732 | 17 | 2.3 | 7.24(3.99-13.14) | <0.0001 | 2.75(1.44-5.24) | 0.002 |
|  | PSM | 732 | 336 | 45.9 | 2.30(1.53-3.39) | <0.0001 | 1.38(0.92-2.07) | 0.12 |
| Shariat,2004 | LVI | 630 | 32 | 5.1 | 6.380(3.629-11.215) | <0.001 | 1.671(0.935-2.986) | 0.083 | LVI | pre-op PSA, PNI, SVI, ECE, PSM, LNI, final Gleason sum |
|  | PNI | 630 | 380 | 60.3 | 2.292(1.368-3.841) | 0.002 | 0.900(0.378-2.172) | 0.815 |
|  | SVI | 630 | 57 | 9 | 6.859(4.320-10.890) | <0.001 | 1.939(1.022-3.676) | 0.043 |
|  | ECE | 630 | 182 | 28.9 | 4.715(2.971-7.483) | <0.001 | 1.721(0.951-3.112) | 0.073 |
|  | LNI | 630 | 10 | 1.6 | 19.441(9.52-39.72) | <0.001 | 1.566(0.932-2.630) | 0.09 |
|  | PSM | 630 | 179 | 28 | 3.322(2.130-5.180) | <0.001 | 1.566(0.932-2.630) | 0.09 |
| Turker,2013 | SVI | 331 | 57 | 17.2 | NA | NA | 1.43(0.63-3.23) | 0.38 | Gleason  pattern | pre-op PSA, tertiary Gleason component, GS, pathological stage, PSM, ECE, SVI, LNI |
|  | ECE | 331 | 122 | 36.9 | NA | NA | 1.25(0.60-2.56) | 0.54 |
|  | PSM | 331 | 80 | 24.2 | NA | NA | 2.28(1.21-4.32) | 0.01 |
|  | LNI | 331 | 13 | 3.9 | NA | NA | 1.15(0.33-4.01) | 0.82 |
| Whittemore,2008 | LVI | 214 | 24 | 11.2 | NA | NA | 2.49(1.09-5.65) | 0.03 | Gleason  pattern | percentage of cancer, pathology stage, PNI, LNI, pre-op PSA, LVI, PSM |
|  | PNI | 214 | NA | NA | NA | NA | 1.75(0.59-5.20) | 0.317 |
|  | LNI | 214 | 5 | 2.3 | NA | NA | 2.54(0.78-8.25) | 0.122 |
|  | PSM | 214 | 114 | 53.3 | NA | NA | 3.50(1.84-6.65) | <0.001 |
| Wolters,2009 | SVI | 344 | 6 | 1.7 | 6.39(1.43-28.49) | 0.015 | 4.20(0.51-34.80) | 0.183 | tumor  volume | age, pathological stage, GS, PSM, tumor volume, SVI |
|  | PSM | 344 | 84 | 23.8 | 4.69(2.76-7.96) | <0.0001 | 3.84(2.15-6.88) | <0.0001 |
| Yee,2010 | LVI | 1298 | 129 | 9.9 | NA | NA | 1.77(1.11-2.82) | 0.017 | LVI | pre-op PSA, SVI, GS, ECE, LNI, PSM |
|  | SVI | 1298 | 91 | 7 | NA | NA | 2.33(1.44-3.75) | <0.001 |
|  | ECE | 1298 | 793 | 61.1 | NA | NA | 3.45(1.91-6.22) | <0.001 |
|  | LNI | 1298 | 94 | 7.2 | NA | NA | 1.38(0.85-2.23) | 0.19 |
|  | PSM | 1298 | 171 | 13 | NA | NA | 1.98(1.28-3.06) | 0.002 |
| Yoshimoto,2008 | LVI | 94 | 26 | 27.7 | NA | NA | 1.645(1.101-2.457) | 0.015 | LVI | RP GS, biopsy GS, PSM, PSA, LVI, clinical stage |
|  | PNI | 122 | 104 | 85.2 | 2.91(1.05-8.04) | 0.0304 | NA | NA |
|  | ECE | 111 | 19 | 17.1 | 3.10(1.67-5.760 | 0.0002 | NA | NA |
|  | PSM | 111 | NA | NA | 2.40(1.41-4.07) | 0.0008 | NA | NA |
| LVI = lymphovascular invasion; PNI = perineural invasion; SVI = seminal vesicle invasion; ECE = extracapular extension; LNI = lymph node involvement; PSM = positive surgical margin; pre-op PSA = preoperative PSA level; GS = Gleason score; RP = radical Prostatectomy; HGPIN = High-grade prostatic intraepithelial neoplasia; pT = pathologic stage; pTI = percentage of tumor involvement; NA=not available; | | | | | | | | | | |
